# Endoplasmic reticulum stress in lung cancer

**DOI:** 10.3389/fonc.2025.1550075

**Published:** 2025-09-05

**Authors:** Donghuan Zhang, Lanlan Lin, Hui Jin, Huajun Mao, Luying Wang, Wenwen Ma, Zhenghong Lao

**Affiliations:** ^1^ Department of Oncology, Deqing People’s Hospital, Huzhou, Zhejiang, China; ^2^ Department of Respiratory Medicine, the Second Affiliated Hospital of Fujian Medical University, Fujian Medical University, Fuzhou, China; ^3^ Department of Urology, Deqing People’s Hospital, Huzhou, Zhejiang, China

**Keywords:** lung cancer, tumor microenvironment, endoplasmic reticulum stress, unfolded protein response, targeted therapy

## Abstract

Endoplasmic reticulum is the primary site of eukaryotic cells involved in biosynthesis, lipid metabolism, glucose metabolism, protein folding and secretion. Multiple factors in the tumor microenvironment may induce the accumulation of unfolded and misfolded proteins in the endoplasmic reticulum and trigger endoplasmic reticulum (ER) stress. Adaptive mechanisms including unfolded protein response (UPR) and endoplasmic reticulum associated degradation (ERAD) are activated in response to ER stress. Previous studies have revealed that ER stress may participate in epithelial mesenchymal transformation, apoptosis, metabolic regulation and drug resistance of lung cancer cells. Herein, we summarized the potential effects and regulatory mechanisms of ER stress on the biological process of lung cancer, which may provide scientific significance and clinical value for elucidating the adaptability of lung cancer cells under stress and developing novel targeted therapies.

## Introduction

Lung cancer is the most prevalent malignant tumor worldwide. The American Cancer Society has reported that lung cancer was the leading cause of cancer-related mortality in 2022, comprising the majority of cancer deaths globally, with around 81% of lung cancer cases being attributed to long-term smoking (1). Additional risk factors include a family history of pulmonary cancer, occupational exposure, previous exposure to infectious pathogens, and various aetiologic factors (2). The universal application of low-dose computed tomography, as an effective modality for lung cancer screening, has significantly improved the detection rate of lung adenocarcinoma (LUAD). However, even patients diagnosed with early-stage and undergo radical surgery, approximately 30%-50% of patients still develop postoperative recurrence, with a 5-year survival rate of less than 60% (3).

Malignant tumor cells and the tumor microenvironment (TME) exhibit reciprocal regulation and adaptation. Due to genomic instability, abnormal metabolism, immune destruction, and alteration of the tumor microenvironment, tumor cells are constantly exposed to exogenous stress (hypoxia, oxidative stress, etc.) and endogenous stress (oncogene activation, tumor suppressor gene inactivation, etc.) that may induce endoplasmic reticulum (ER) stress (4). Stress may trigger autophagic apoptosis in tumor cells (5), while sustained ER stress contributes to the therapeutic resistance. This is largely attributed to high plasticity and adaptive capacity of tumor cells that enable them to survive and even convert the stress into a drive for self-renewal (6). Studies have demonstrated that ER stress is closely associated with immune regulation and drug resistance in lung cancer. The identification of stress-related signaling targets and regulatory mechanisms is of paramount scientific and clinical significance for elucidating the adaptive capacity of lung cancer cells under stress and developing novel targeted therapies.

## UPR and ERAD

The endoplasmic reticulum is a crucial inner membrane structure in eukaryotic cells that plays a fundamental role in various cellular processes, including biosynthesis, lipid and glucose metabolism, protein folding, and secretion of over 30% of intracellular proteins (7). Previous studies have indicated that ER may serve as a “largest processing plant” in the cell, precisely regulating protein folding and modification processes, as well as cellular signals transduction. However, various physiological and pathological factors in the ER may cause disruption of calcium ion homeostasis and protein misfolding in the lumen, leading to an imbalance in ER homeostasis. The imbalance can cause unfolded and misfolded proteins to accumulate in the ER lumen and trigger the ER stress response (8). Under stress conditions, cells initiate a series of cascade response pathways, including the unfolded protein response (UPR) and endoplasmic reticulum-associated degradation (ERAD), to adapt to protein folding alterations (9).

The UPR is an interconnected signaling pathway that involves three transmembrane proteins on the ER membrane, namely inositol-requiring enzyme 1α (IRE1α), PKR-like ER kinase (PERK), and activating transcription factor 6 (ATF6). The primary function of UPR is to restore ER homeostasis by adapting to changes in protein folding within the ER (10). Glucose-regulated proteins 78 (GRP78), an ER resident chaperone also referred to as binding immunoglobulin protein (BiP), may serve as central receptors for ER stress (11). In the resting state of the ER, the luminal structural domains of ATF6, PERK, and IRE1α are bound with the chaperone protein GRP78/BiP in an inactive state. While accumulation of misfolded proteins in the ER lumen may trigger ER stress and contribute to the GRP78/BiP complex dissociating from the sensors, leading to the activation of UPR pathway to restore ER homeostasis and protect the cell from disruption (12).

ERAD is a crucial protein quality control system for the degradation of misfolded proteins in the endoplasmic reticulum. In mammals, ERAD substrates are recognized through the synergistic cooperation of multiple proteins (13). Once identified, ubiquitin can be transferred to E2 enzymes via the ubiquitin-activating enzyme, and transferred to substrate proteins by ubiquitin ligases. Subsequently, ERAD substrates are translocated through the retro-translocation channel and released into the cytoplasm for degradation by the 26S proteasome (14). Derlins are a family of multi-transmembrane proteins in the endoplasmic reticulum that share structural composition and physicochemical features like rhodopsin superfamily, and act in synergy with other proteins to regulate ERAD substrate recognition (15). For example, Derlin-1, a six-transmembrane protein, can form homo- or hetero-oligomers with homologs Derlin-2 and Derlin-3 to facilitate the retro-translocation of full-length defective proteins (16). Additionally, the ERAD-associated rhomboid proteins RHBDL4 cleaves specific membrane substrates into fragments, and subsequently retro-translocated to the cytoplasm for degradation by the proteasome.

To further clarify ERAD’s mechanistic core, the E3 ubiquitin ligase complexes Hrd1, gp78, and SEL1L-Hrd1synergize through modular specialization to mediate substrate recognition, ubiquitination, and retro-translocation: The conserved Hrd1 complex targets ERAD-L (misfolded luminal glycoproteins, via EDEM-dependent demannosylation and OS-9/XTP3B glycan sensing) and ERAD-M (membrane proteins with misfolded transmembrane domains, via Hrd1/Derlin-1 hydrophobic detection), catalyzing K48-linked polyubiquitination and driving retro-translocation via a Derlin-1/Hrd1 membrane-distorting channel coupled to the p97 ATPase (17). The specialized gp78 complex focuses on ERAD-M and ERAD-C, recognizing cytosolic hydrophobic motifs and recruiting endocytic machinery for mislocalized substrates; it generates mixed K48/K63 ubiquitin chain, with activity enhanced by mTORC1-mediated phosphorylation, and relies on a p97-binding VBM motif and helix-unfolding of transmembrane domains for translocation (17, 18). The mammalian SEL1L-Hrd1 hub uses SEL1L’s leucine-rich repeats to stabilize Hrd1 and screen for conformational defects, allosterically regulating Hrd1’s ubiquitination and integrating Derlin-1’s hydrophobic groove, p97 membrane anchoring, and dynamic deubiquitination (e.g., USP19) for fine-tuned retro-translocation (19). Collectively, these complexes couple substrate-specific recognition, ubiquitin chain diversity, and p97-dependent translocation to link ERAD with autophagy and metabolic stress responses, underpinning ER proteostasis and disease pathogenesis. Previous studies have demonstrated that ERAD regulates tumor immunomodulation, therapeutic resistance, and cell survival (20, 21). Targeting essential regulatory proteins of the ERAD system to regulate the survival and apoptosis of tumor cells may be a potentially attractive option for cancer therapy.

## Drivers of ER stress in tumor microenvironment

**Figure 1:**
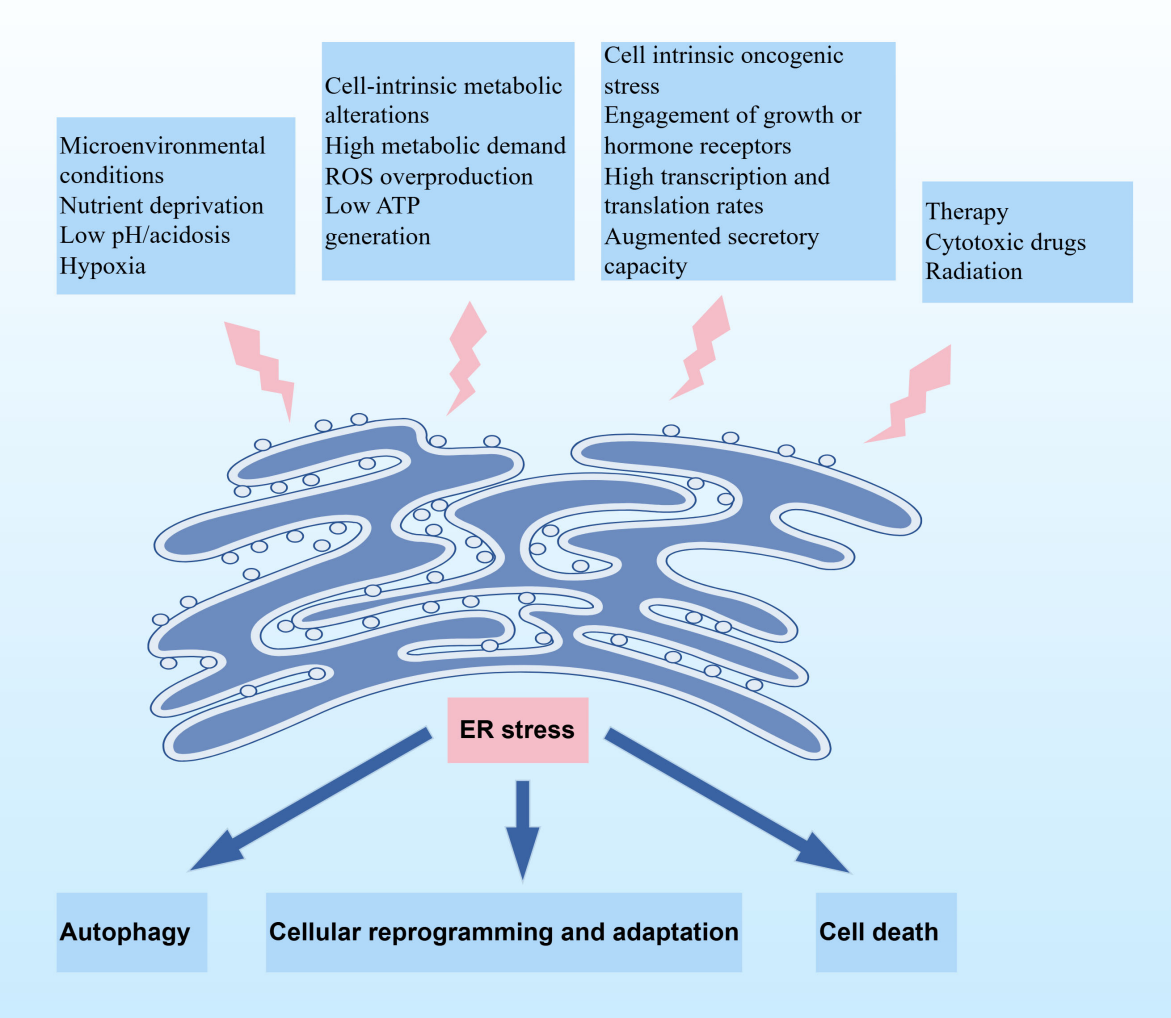
Schematic illustration depicting the induction of ER stress and its downstream cellular fates. Upstream factors triggering ER stress include microenvironmental conditions, cell-intrinsic metabolic alterations, cell-intrinsic oncogenic stress, and therapeutic interventions. ER stress elicits three principal outcomes: autophagy, cellular reprogramming and adaptation, or cell death, depending on the stress intensity and cellular context.

Multiple stressors in the tumor microenvironment, including hypoxia, oxidative stress, nutritional deficiencies, and acidosis, can disrupt the proper folding of intracellular proteins and trigger the accumulation of unfolded and misfolded proteins in the endoplasmic reticulum. To address the challenge, the adaptive mechanisms, such as UPR and ERAD, are activated to maintain proper protein homeostasis within the endoplasmic reticulum (**Figure 1**).

## Hypoxia

The imbalance between high oxygen consumption and abnormal vascularization of malignant tumors in the microenvironment results in the generation of low oxygen regions in the center of solid tumors. Hypoxia, an essential characteristic of the microenvironment, disrupts the homeostasis of the endoplasmic reticulum by impairing post-translational modification processes such as glycosylation and disulfide bond formation (22). Disulfide bond formation is dependent on molecular oxygen, which is the ultimate acceptor for electron transfer during the process and is crucial for post-translational protein folding and isomerization (23). ERO-1α, a regulatory oxidoreductase in the endoplasmic reticulum, synergizes with protein disulfide isomerase (PDI) and peroxiredoxin 4 (PRX4) to facilitate disulfide bond formation and protein folding (24, 25). The interaction between ERO-1α and PDI constitute the pivotal oxidative folding pathway in the ER. However, limited ATP production under hypoxic conditions may impair the oxygen-dependent protein folding process, leading to the accumulation of unfolded or misfolded proteins in the endoplasmic reticulum lumen.

## Oxidative stress

Reactive oxygen species (ROS) are oxygen radicals in living organisms, including oxygen and highly reactive oxygen-containing molecules (e.g., superoxide anions, hydrogen peroxides, and free radicals). ROS are formed continuously in mitochondria by electron leakage from the respiratory chains, and contribute to cellular signaling, regulation of gene expression, and intracellular calcium levels (26, 27). Prior studies have shown that during the UPR, PDI synthesis is markedly increased to promote the formation of disulfide bonds in misfolded proteins, accompanied by the production of electrons (28). Simultaneously, intracellular reduced glutathione (GSH), with oxygen as the final electron acceptor, is oxidized to oxidized glutathione (GSSG) with the production of ROS (29). The redox imbalance caused by GSH depletion in the microenvironment is the key factor triggering ER stress in lung cancer cells. Elevated ROS levels caused by GSH deficiency exacerbate ER stress by activating the iNOS/ATF4/DDIT3 pathway, while promoting mitochondria-endoplasmic reticulum interactions to further amplify stress signals (30, 31). Disruption of redox balance between GSH and GSSG may induce ER stress after oxidative stress. In addition, the accumulation of unfolded proteins in the endoplasmic reticulum promotes Ca^2+^ leakage from the ER lumen into the cytosol, which stimulates mitochondrial tricarboxylic acid (TCA) cycle and oxidative phosphorylation, leading to the overproduction of ROS in mitochondria. Oxidative stress resulting from excessive ROS can disrupt the homeostasis of endoplasmic reticulum and causes the formation of unfolded protein deposits.

## Acidosis

Metabolic reprogramming is a hallmark of cancer, wherein tumor cells reprogram nutrient utilization to meet the cellular demands for bioenergetics and biosynthesis (32). Compared with normal cells, tumor cells may utilize the aerobic glycolysis pathway to adapt to low glucose levels, for example, they take up more glucose and convert glucose into pyruvate by aerobic glycolysis which ultimately generates adenosine with the inhibition of downstream glycolysis steps catalyzed by pyruvate kinase and pyruvate dehydrogenase. Such a phenomenon is referred to as “Warburg effect”, which facilitates the survival and growth of tumor cells under hypoxic conditions (33). The increased acid production in tumor microenvironment activates the acid-sensing ion channel 1α (ASIC1α) on the cell surface, leading to the activation of calcium ion channels and subsequent calcium overload. Calcium overload is a crucial initiator of ER stress (34). Calcium accumulation in mitochondria causes uncoupling of electron transport and obstructs ATP generation. Owing to insufficient cellular energy supply, an amount of the unfolded proteins may accumulate in the endoplasmic reticulum and further triggering calcium ion release, exacerbating intracellular calcium overload and inducing ER stress (35).

## Glutamine deficiency

Glutamine serves as a vital precursor for the synthesis of proteins, nucleotides, and other macromolecules in mammals. Additionally, it generates nicotinamide adenine dinucleotide phosphate (NADPH) and GSH, which help maintain redox homeostasis and defend against free radicals (36). There is an extensive consumption of glutamine in the tumor microenvironment. The “Warburg” effect suggests that tumor cells preferentially provide energy by means of glycolysis, even under conditions of sufficient oxygen. This preference for glycolysis affects the mitochondrial energy supply. To maintain mitochondrial energy supply, tumor cells rely on the glutamine transporter to uptake glutamine and replenish the metabolites of TCA, providing the necessary substrates for overactivated glycolysis and oxidative phosphorylation reactions (37). Prior studies have shown that tumors consume glutamine at 5–10 times the rate of normal cells, highlighting the dependence on glutamine (38). However, the reprogramming of glutamine metabolism in tumors disrupts the balance of glutathione and NADPH, thereby perturbing intracellular redox homeostasis.

In addition, the deficiencies of glutamine and glucose in the microenvironment can disrupt the hexosamine biosynthesis pathway (HBP) (39). The HBP is an essential branch of intracellular glucose metabolism, integrating glucose metabolism, glutamine breakdown, fatty acid metabolism, and nucleotide metabolism. The pathway utilizes glucose, glutamine, acetyl-CoA, and UTP to produce uridine diphosphate N-acetylglucosamine (UDP-GlcNAc), which is an essential donor for the biosynthesis of polysaccharides, proteoglycans, glycolipids, and O-GlcNAc modifications (40, 41). The impact of the HBP is closely linked to the content and destination of UDP-GlcNAc. Studies have shown that glycosylation is not only a major post-translational protein modification mechanism, but also critical for maintaining protein structure and activity. O-GlcNAc is the primary source of glycosylation and folding of modified endoplasmic reticulum proteins (42). Glucose and glutamine deficiency in the TME may alter the production of glutathione and promote accumulation of unfolded proteins in the endoplasmic reticulum, thereby increasing ER stress.

## ER stress in lung cancer

**Figure 2:**
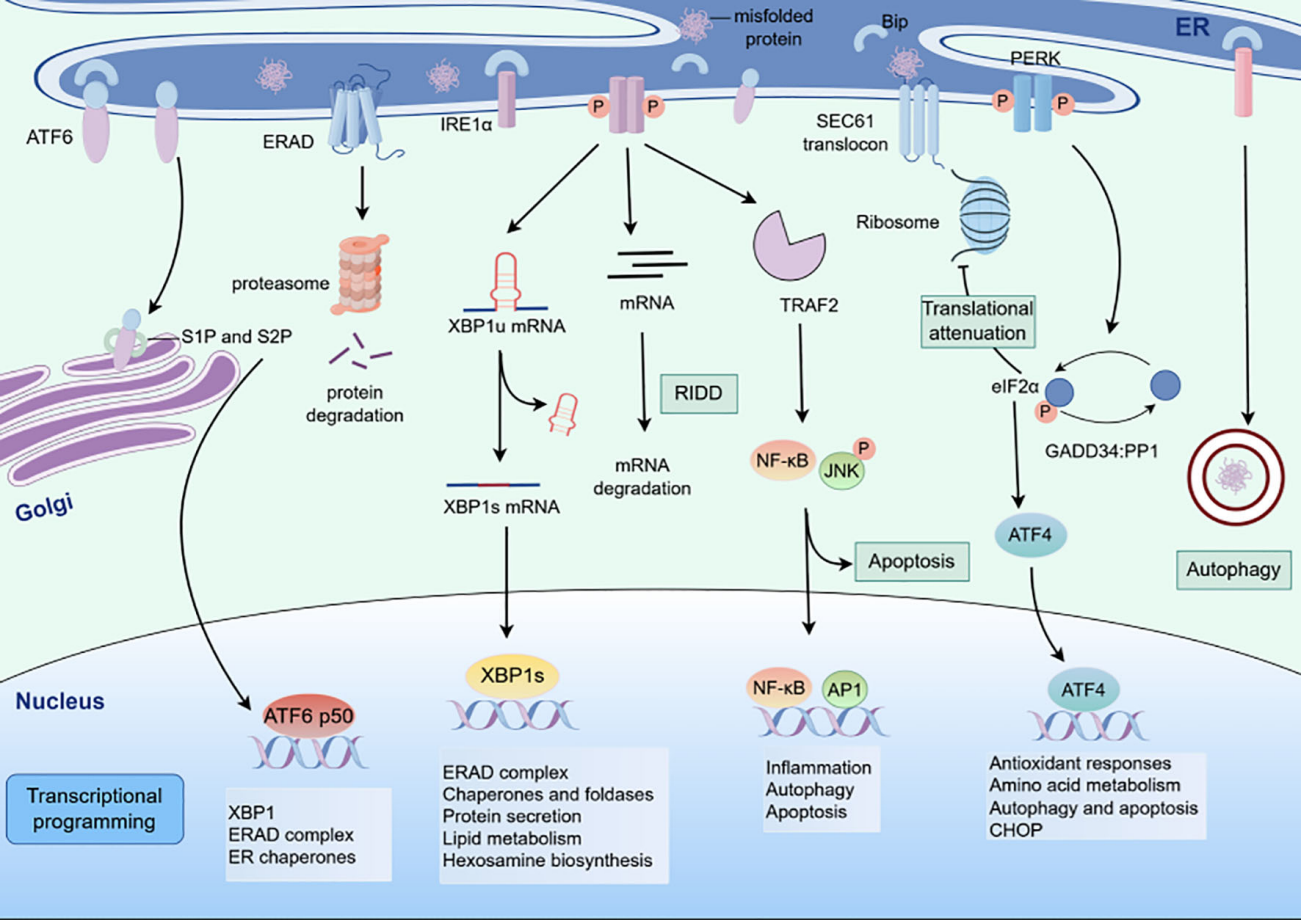
Schematic of ER stress - induced unfolded protein response (UPR) signaling pathways. The three canonical UPR signaling cascades activated by endoplasmic reticulum (ER) stress. When misfolded proteins accumulate in the ER, three ER - resident sensors (IRE1α, PERK, and ATF6) initiate distinct signaling branches.

The diverse drivers of ER stress within the TME do not act in isolation but instead converge on lung cancer cells to elicit context-specific perturbations in protein homeostasis. These TME-derived stressors directly impinge on the ER of lung cancer cells, activating UPR pathways that serve as critical nodes linking extracellular cues to intracellular phenotypic adaptations. Specifically, the cumulative effects of TME-induced ER stress reprogram key cellular processes in lung cancer, including the induction of epithelial-mesenchymal transition (EMT) to enhance metastatic potential, the regulation of apoptotic and autophagic machinery to balance survival and cell death, and the modulation of drug resistance mechanisms to evade therapeutic pressure (**Figure 2**).

## ER stress and EMT

EMT refers to a process in which epithelial cells lose their polarity, cytoskeletal structure, and intercellular adhesion, acquiring the migratory characteristics of mesenchymal cells (43). Recent studies have shown that ER stress can induce EMT in lung cancer cells. Tunicamycin (TM) and thapsigargin (TG), as ER stress inducers, can disrupt calcium homeostasis, redox balance, and N-glycan synthesis, leading to non-specific activation of the UPR. The activated UPR then induces EMT in a Smad2/3- and Src-dependent manner (44). The ubiquinone protein UBQLN1 plays a critical role in the ubiquitin-proteasome pathway (UPP), transporting polyubiquitin proteins to the proteasome and assisting the ERAD process in clearing unfolded proteins (45, 46). Shah et al. reported that the UBQLN1 deficiency in lung cancer cells promotes cytoskeleton formation, increases the expression of stromal phenotype-related proteins such as Vimentin, Snail, and ZEB1, and inhibits the expression of epithelial phenotypic markers such as E-Cadherin and Claudin1, ultimately regulating the EMT in lung cancer cells (47).

Abnormally activated EMT can modulate the sensitivity of tumor cells to ER stress. The expression of EMT-related genes is strongly associated with the extracellular matrix (ECM) and PERK-eIF2α pathway. Lung cancer cells undergoing EMT may remodel the ECM by secreting matrix proteases and scaffold proteins, and activate the PERK-eIF2α axis to enhance their sensitivity to ER stress. Furthermore, maintaining endoplasmic reticulum homeostasis via the PERK-eIF2α signaling pathway is also essential for EMT-mediated cell invasion and metastasis.

## ER stress and apoptosis

The ER stress response is a protective cellular mechanism that helps to alleviate the accumulation of unfolded proteins and attenuate endoplasmic reticulum dysfunction. However, persistent and excessive stress can trigger intracellular apoptotic signals and promote apoptosis (48). ER stress induces apoptosis through the activation of multiple pathways, including the c/EBP homologous protein (CHOP), Caspase-12, and JNK pathways. The PERK-eIF2α-ATF4 signaling pathway is the primary pathway that facilitates CHOP protein expression. During ER stress, PERK dissociates from GRP78/BiP and activated through phosphorylation. Activated PERK further promotes ATF4 translation, which cooperates with ATF6 and XBP-1 to enter the nucleus and bind to the CHOP promoter to activate its transcriptional level (49). Overexpressed CHOP induces apoptosis by downregulating the anti-apoptotic protein Bcl-2 and upregulating the pro-apoptotic protein Bax, ultimately triggering apoptosis through Caspase-3 activation (50). Gan et al. reported that in tunicamycin-induced ER stress, mutant p53 lung cancer cells undergo apoptosis and autophagy by increasing the expression of CHOP, GRP78, IRE1α, and Caspase-3 (51). Besides, rolapitant may trigger the ER stress-CHOP-DR5 signaling pathway by targeting the OTUD3-GRP78 axis, enhancing TRAIL induced apoptosis in lung cancer cells (52).

The apoptosis of lung cancer cells mediated by JNK is predominantly driven by the IRE1α signaling pathway. Upon ER stress, IRE1α recruits TRAF2 and ASK1 to form the IRE1α-TRAF2-ASK1 complex, which then phosphorylates apoptosis regulatory kinases, resulting in the activation of pro-apoptotic IRE1-JNK signaling axis. JNK phosphorylation can activate the activities of pro-apoptotic proteins BIM and BMF, thereby enhancing their susceptibility to apoptosis (53, 54). Additionally, the IRE1α-TRAF2 complex triggers the dissociation of procaspase-12 from TRAF2 and activates Caspase-12. Caspase-12 further cleaves and activates Caspase-9, which in turn activates Caspase-3 to induce apoptosis (55). Zhang et al. have demonstrated that CSTMP-induced ER stress-related apoptosis in lung adenocarcinoma mainly depends on the activation of Caspase-12, Caspase-4, and the IRE1α-TRAF2-ASK1-JNK signaling pathway (56).

## ER stress and autophagy

ER stress and autophagy form a complex interplay network. Autophagy alleviates ER stress by clearing misfolded proteins to sustain tumor cell survival, for instance, Sestrin2, a stress-inducible protein, dualistically suppresses ER stress via PERK pathway inhibition while activating AMPK-mediated autophagy, effectively curbing apoptosis in lung cancer cells (57), whereas excessive autophagy may trigger apoptosis or ferroptosis. Lai et al. have demonstrated that the marine compound Crassolide triggers unresolved ER stress, leading to autophagosome overload and subsequent G2/M phase arrest via ATF4-CHOP-dependent ferroptosis in lung adenocarcinoma. Mechanistically, Crassolide disrupts ER-mitochondria contact sites, amplifying mitochondrial ROS and lipid peroxidation (58, 59).

The dynamic equilibrium between ER stress and autophagy is orchestrated by the unfolded protein response. PERK and IRE1α branches upregulate autophagy adaptors (LC3-II, Beclin1) through transcriptional activation of ER chaperones (GRP78, CHOP), enabling transient proteotoxic stress resolution (60, 61). However, persistent ER stress overwhelms this adaptive response, shifting autophagy toward apoptosis via calcium-mediated caspase-12 activation (62). Pharmacologically, the ER stress inhibitor 4-PBA attenuates both autophagy flux and apoptosis, rescuing chemotherapy-induced normal tissue damage (58, 60). Furthermore, ER stress-mediated autophagy enhancement serves as a critical mechanism of chemoresistance, and autophagy inhibition (e.g., chloroquine) can reverse drug resistance while amplifying ER stress-dependent apoptosis (63). Collectively, targeting the ERS-autophagy regulatory network provides novel therapeutic avenues for lung cancer treatment.

## ER stress and drug resistance

ER stress can directly regulate the drug resistance in lung cancer cells. The molecular chaperone GRP78 mediates cisplatin resistance in lung adenocarcinoma endoplasmic reticulum stress tolerant (ERST) through the activation of the Akt cascade (64). GRP78 can also localize on the surface of tumor cells as surface GRP78 (sGRP78), which is preferentially overexpressed in invasive, metastatic, and chemotherapy-resistant cancers (65). sGRP78, as a receptor of multiple signal pathways on the cell surface, transmits signals to endow cancer cells with stem cells properties and epithelial-mesenchymal transition ability, and mediates gefitinib resistance (66, 67). The plasminogen Kringle5 domain may bind to sGRP78 of endothelia and tumor cells, reducing the proliferation and colony formation of non-small cell lung cancer (NSCLC) cells, and alleviating radiotherapy resistance (68).

CHOP is also an indispensable part of the ER stress response, and increased expression of CHOP enhances sensitivity to cisplatin in lung cancer cells (69, 70). Wang et al. have shown that CHOP regulates cisplatin resistance in NSCLC cells by promoting the expression of apoptotic proteins and inhibiting the Bcl-2/JNK signaling pathway (71). Cisplatin has also been demonstrated to induce ER stress in lung cancer cells through the PERK/IRE1 signaling pathway. Inhibition of ER stress with the ER stress inhibitors 4-phenylbutyric acid (4-PBA) or tauroursodeoxycholic acid sodium salt (TUDCA) has been found to increase the sensitivity of lung cancer cells to cisplatin (72).

## ER stress and immune regulation

The UPR pathways-PERK, IRE1α, and ATF6 may modulate immune cell function within the tumor microenvironment. For instance, PERK activation in cancer cells upregulates PD-L1 expression, impairing cytotoxic T-cell activity and fostering immune evasion (73) Concurrently, ER stress in dendritic cells (DCs) disrupts antigen presentation by downregulating MHC class I molecules, as shown in preclinical lung adenocarcinoma models (74).

Paradoxically, ER stress may also trigger tumor immunogenic cell death (ICD). The ICD process induces tumor cell death under stress, resulting in the exposure of tumor-associated antigens that activate cytotoxic T cells and trigger anti-tumor immune responses (75). Calreticulin (CRT), a damage-associated molecular pattern (DAMP) molecule, is released and translocated from the endoplasmic reticulum to the tumor cell membrane during ICD. CRT may bind to low-density lipoprotein receptor on the surface of DCs, promoting the phagocytosis of tumor cells by DCs (76, 77). A retrospective analysis showed that high expression of CRT on tumor cells was strongly correlated with eIF2α phosphorylation and mature DC infiltration, which had a positive impact on the clinical prognosis of NSCLC patients (78). Additionally, Wang et al. reported that Ir1 anchored to the endoplasmic reticulum activates the ER stress response, contributing to ICD activation via CD8^+^ T cell-mediated immune responses and Foxp3^+^ T cell exhaustion, ultimately producing long-term anti-tumor immunity (79).

ER stress further amplifies immunosuppressive signals by recruiting regulatory T cells (Tregs). Notably, ER stress sustains Treg stability through molecules such as transmembrane protein TMED4. TMED4 deficiency destabilizes Foxp3 expression, impairing Treg immunosuppressive capacity and thereby enhancing antitumor immunity (80). Additionally, chronic ER stress fosters a metabolic niche favoring Treg dominance by suppressing mitochondrial respiration and cytotoxicity in CD8^+^ T cells, indirectly amplifying Treg-mediated immunosuppression (81). Furthermore, ER stress-activated tumor cells secrete immunosuppressive factors like sphingosine-1-phosphate (S1P), forming an “ER stress-S1P-CAMP axis” that drives Treg expansion and establishes an immunosuppressive TME (82).

In addition, ER stress can promote the polarization of M1 macrophage or inhibit the polarization of M2 macrophage by activating pathways such as PERK and IRE1α, thereby affecting the progression of lung cancer (83). Zhou et al. have also found that Piperlongumine (PL) inhibits the polarization of tumor-associated macrophages into the M2 phenotype by inducing ER stress in lung cancer cells, thereby reducing tumor cell migration. *In vitro* experiments have confirmed that the ER stress inhibitor 4-PBA can reverse the effects of PL, indicating the critical role of ER stress in macrophage polarization (84).

## ER stress signal transduction pathway

**Figure 3:**
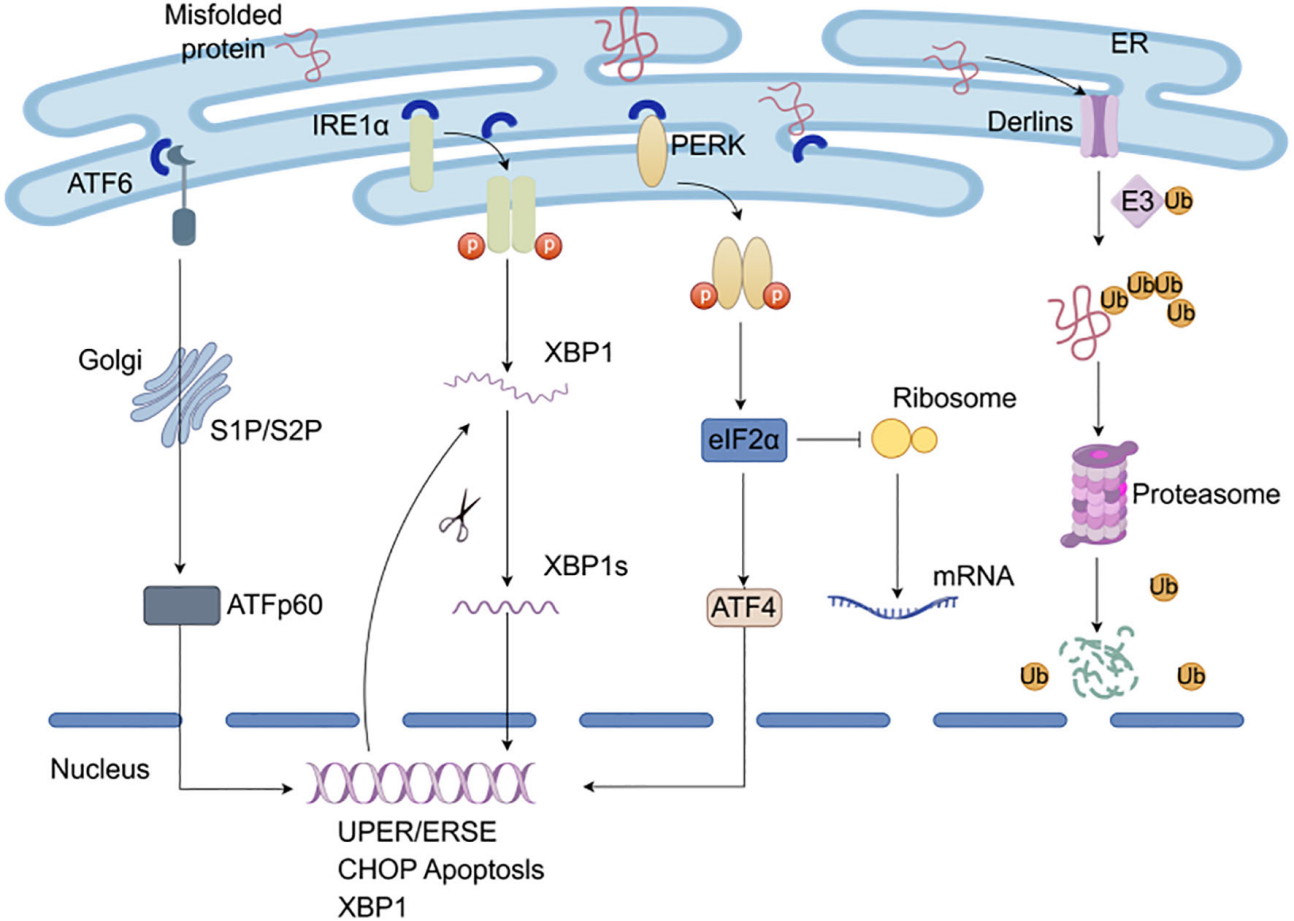
Protein quality control system. The UPR responsible for restoring ER homeostasis by adapting to changes in protein folding within the ER through ER stress sensors PERK, IRE1α, and ATF6. Misfolded or unfolded proteins accumulate and compete with the stress sensors for binding to chaperones and then activate downstream signaling pathways. In ERAD, misfolded substrates are recognized and shuttled from the ER lumen into the cytosol for degradation by the proteasome.

The UPR primarily inhibits protein synthesis through ER stress sensors PERK, IRE1α, and ATF6 (**Figure 3**). In physiological conditions, the ER stress sensors are bound to the molecular chaperone GRP78/Bip. While when cells are exposed to internal and external stressors, misfolded or unfolded proteins accumulate and compete with the stress sensors for binding to GRP78, as a result PERK, IRE1α, and ATF6 dissociate from GRP78 and activate downstream signaling pathways.

## PERK-eIF2α signal pathway

PERK is a type I transmembrane protein located on the endoplasmic reticulum, with its N-terminal stress-sensing domain binding to GRP78 to prevent dimerization. When PERK dissociates from GRP78, its C-terminal serine/threonine protein kinase catalytic domain is activated through autophosphorylation. The activated PERK further phosphorylates downstream eIF2α, and the phosphorylated eIF2α inhibits the assembly of 80S ribosomal subunits and terminates mRNA translation, thereby effectively suppressing the rate of protein synthesis (85). Activated eIF2α may upregulate the translation of ATF4 and the expression of growth arrest and DNA damage-inducible 34 (GADD34) and CHOP. When excessive ER stress triggers overactivation of the unfolded protein response, it induces sustained phosphorylation-mediated inhibition of eIF2α, under which condition ATF4 exerts pro-apoptotic effects. Although the reduction in protein synthesis under ER stress may grant stressed cells additional time to resolve aberrant protein accumulation, prolonged PERK signaling–by persistently blocking protein translation–becomes detrimental to survival. To counteract this, ATF4 transcriptionally upregulates growth arrest and GADD34, a regulatory subunit of protein phosphatase 1 (PP1), which dephosphorylates p-eIF2α. This restores the eIF2α-GTP-Met-tRNA ternary complex, thereby resuming mRNA translation and preserving cellular homeostasis (86). However, if ER stress remains unresolved, persistent PERK activation ultimately upregulates CHOP, a key mediator of apoptosis that disrupts redox balance and mitochondrial integrity. The highly expressed CHOP downregulates anti-apoptotic proteins (e.g., Bcl2) while upregulating pro-apoptotic members (e.g., Bax, Bim), thereby driving apoptotic cell death, ultimately initiating the process of ER stress-mediated programmed cell death (87).

## IRE1α-XBP1 signal pathway

IRE1 is the most highly conserved UPR sensor in eukaryotic cells and possesses both serine/threonine protein kinase activity and endonuclease activity. When cells undergo ER stress, the IRE1α dissociates from GRP78/Bip and undergoes autophosphorylation and dimerization, which activates its endonuclease activity to splice and modify the XBP1 mRNA precursor. The process involved in the removal of introns and permission of the XBP1 translation. Subsequently, spliced XBP1 enters the nucleus to mediate ERAD, protein folding, and endoplasmic reticulum membrane expansion (88). Conversely, unresolved and persistent ER stress leads to sustained, high-level dimerization and autophosphorylation of IRE1α. This enhances the ribonuclease activity of IRE1α, primarily augmenting its splicing efficiency toward XBP1 mRNA. More critically, it concurrently reduces the substrate specificity of IRE1α’s ribonuclease domain, enabling nonspecific degradation of hundreds of mRNAs translated on the ER membrane–a process termed regulated IRE1α-dependent decay (RIDD) (89). While RIDD transiently alleviates ER protein-folding burden by degrading secretory pathway transcripts, the indiscriminate cleavage of ER-associated mRNAs ultimately depletes critical ER-resident enzymes and structural components (e.g., chaperones, translocon subunits), exacerbating ER dysfunction (90). Under severe ER stress, RIDD not only disrupts ER protein processing but also drives ER membrane destabilization and Caspase-12-mediated apoptosis, positioning it as a double-edged sword in cellular stress adaptation.

## ATF6 signal pathway

ATF6 is a type II transmembrane transcription factor located on the endoplasmic reticulum membrane, consisting of two subunits (ATF6α and ATF6β) and containing a basic leucine zipper domain (91). ATF6 is packaged into transport vesicles and transported from the ER to the Golgi apparatus during ER stress. In the Golgi, ATF6 is cleaved into transcription factor ATF6p50 by proteolytic enzymes S1P and S2P. The cleaved ATF6p50 migrates to the nucleus to activate unfolded protein response elements (UPREs) or ER stress elements (ERSEs) and promote the refolding of ERAD-related proteins (92). Additionally, ATF6 may activate the transcription of CHOP to induce apoptosis and enhance the expression of unspliced XBP1, thereby linking to the IRE1α pathway (93).

## Drugs targeting ER stress

The PERK, IRE1α, and ATF6 signaling pathways play a crucial role in the immune regulation, invasion, and migration of tumor cells. To date, Existing studies have identified inhibitors targeting the UPR signaling pathway show the promising anti-tumor potential in lung cancer. Nonetheless, the efficacy and clinical translation of these drugs require verification through further basic and preclinical trials (**Table 1**).

**Table 1:** Medications targeting endoplasmic reticulum stress and applications.

Specific molecule	Inhibitors	Mechanism	Research progress	Application
IRE1α	Kira6, Kira7, Kira8, STF-083010, 4μ8C, PAIR2, BI09, MKC9989, MKC8866, MKC3946 and 3,6-DMAD hydrochloride	Inhibit IRE1α RNase activity, reduce XBP1 splicing, alleviate UPR, and decrease pro-inflammatory cytokine release	Preclinical studies show protective effects in neurodegenerative diseases and diabetic nephropathy in animal models. In addition, the inhibitors may block Osimertinib resistance and endoplasmic reticulum stress-related apoptosis in non-small cell lung cancer cells	Lung cancer, breast cancer, prostate cancer, melanoma, lymphoma, multiple myeloma, neurodegenerative diseases, metabolic diseases
	APY29 and IRE1α kinase-IN-5	ATP competitive inhibitor, inhibition of IRE1 α autophosphorylation by binding to ATP binding sites		
PERK	PERK-IN-2, PERK-IN-3, PERK-IN-4, PERK-IN-5, GSK2606414, GSK2656157, AMG PERK 44 and ISRIB	Inhibit PERK kinase activity, reduce eIF2α phosphorylation, restore protein synthesis, and avoid excessive apoptosis	Animal studies show efficacy in pancreatic cancer and retinopathy; requires balancing protein synthesis and misfolded protein accumulation risks	COPD, breast cancer, retinopathy, autoimmune disease
ATF6	Melatonin, Melatonin D-3, Melatonin D-5, and Melatonin D-7	Inhibit ATF6 activation, alleviate endoplasmic reticulum stress state through concentration dependent approach	Reduces apoptosis in myocardial ischemia-reperfusion injury models, research focuses on cardiovascular diseases	Cardiovascular diseases, liver injury, glioma, pulmonary fibrosis, lung cancer

## PERK inhibitor

The PERK signaling pathway is activated in the cytoplasm in response to ER stress. Several highly efficient and selective PERK inhibitors with oral bioavailability have been identified, including PERK-IN-2, PERK-IN-3, PERK-IN-4, and PERK-IN-5 (94). GSK2606414 and GSK2656157 are ATP-competitive PERK inhibitors with high selectivity and cell permeability, which inhibit PERK Thr980 phosphorylation in a dose-dependent manner (95, 96). Studies have shown that GSK2606414 can effectively inhibit the ER stress response of lung cancer cells and slow the growth of lung cancer allograft models in mice (97). Moreover, GSK2606414 inhibited nitrofurazone (NFZ)-induced elevation of ROS and Ca^2+^ levels, blocked the activation of the PERK-ATF4-CHOP signaling pathway, and consequently suppressed NFZ-induced apoptosis in NSCLC cells (98), which suggests that GSK2606414 may influence tumor cell fate through regulation of oxidative stress and calcium homeostasis. Additionally, GSK2606414 demonstrates potential in regulating the inflammatory microenvironment of lung cancer by inhibiting PERK, which concurrently attenuates both NF-κB-mediated inflammatory responses and apoptosis (99). Other highly selective PERK inhibitors include AMGPERK44 and ISRIB, which can effectively reverse eIF2α phosphorylation (100). ISRIB increases DUSP6 levels to reduce TG-induced PERK/p-eIF2α activation and inhibit chemotherapy resistance of KRAS-driven lung cancer cells (101). Moreover, ISRIB can regulate lung cancer immunotherapy by inhibiting PD-L1 expression (102, 103). However, there are few reports on the research of AMGPERK44 in lung cancer. Currently, only studies have shown that the combination of AMGPERK44 and the Ref-1 inhibitor can significantly enhance the killing effect on pancreatic cancer cells and cancer-associated fibroblasts (CAFs), especially in the 3D co-culture model (104).

## IRE1α-XBP1 inhibitor

IRE1α inhibitors suppress the activation of IRE1α by targeting both its ribonuclease and serine/threonine kinase activities. Several compounds have been identified as IRE1α RNase inhibitors, including Kira6, Kira7, Kira8, STF-083010, 4μ8C, PAIR2, BI09, MKC9989, MKC8866, and MKC3946. These compounds have been extensively investigated for the potential in treating various types of cancer, including lung cancer, breast cancer, prostate cancer, melanoma, lymphoma, and multiple myeloma (105). STF-083010 inhibits IRE1 endonuclease activity, blocks osimertinib resistance in NSCLC cells induced by IRE1 signal transduction (106), and reverses ER stress-induced apoptosis through the PERK/IRE1α/ATF6 pathway (107). In addition, ATP-competitive inhibitors APY29 and IRE1α kinase-IN-5 are specific allosteric regulators of IRE1α kinase activity, which inhibits the autophosphorylation of IRE1α by combining its ATP binding sites (108). Sunitinib, a multi-target tyrosine kinase inhibitor, and its deuterated derivative, sunitinib-D10, effectively restrain the phosphorylation of IRE1α by competing with ATP binding and subsequently inhibiting its autophosphorylation and RNase activation (109).

3,6-DMAD hydrochloride and toyocamycin have been identified to reverse the ER stress by the IRE1α-XBP1 pathway. 3,6-DMAD hydrochloride, an acridine derivative, represses the IRE1α-XBP1 pathway (110, 111). It inhibits IRE1α oligomerization, RNase activity, and XBP1 splicing *in vivo* and has been demonstrated to prevent the growth of multiple myeloma (111, 112). However, there have been limited reports on the study of 3,6-DMAD hydrochloride in lung cancer at present. Toyocamycin, an adenosine analogue produced by Streptomyces, is an inhibitor of XBP1 (113). It can block RNA synthesis and ribosome function, inhibit XBP1 mRNA cleavage, and reduce the activity of tumor cells that have been activated by IRE1α (114).

## ATF6 pathway inhibitor

There are several inhibitors of the ATF6 pathway, including melatonin and its deuterated derivatives, melatonin D-3, melatonin D-5, and melatonin D-7 (115). Melatonin is a hormone secreted by the pineal gland that regulates the circadian clock and primarily binds to melatonin receptors MT1 and MT2 (116). As a novel selective ATF6 inhibitor, melatonin can reduce the ER stress in a concentration-dependent manner and prevent glioma cell death caused by excessive ER stress activation (117). Studies have also indicated that melatonin alleviates ER stress and bleomycin-induced EMT in lung fibroblasts by inhibiting ATF6 and α-SMA (118). Notably, melatonin has ever demonstrated protective effects against lung cancer in multiple animal models. For example, in a passive smoking-induced lung injury model, it reduces lung tissue damage, apoptosis, and inflammatory responses by decreasing ATF6 activation while downregulating the expression of lung cancer-related genes (such as VEGF, CYP1A1, and CYP1B1) (119); in the Lewis lung cancer (LLC) mouse model, it significantly inhibits tumor growth by suppressing the NLRP3 inflammasome and ATF6-related pathways, accompanied by reduced expression of pro-angiogenic and lymphangiogenic markers in tumor tissues (120). Additionally, the combination of melatonin and ortho-topolin riboside (oTR) has exerted synergistic anti-tumor effects by regulating metabolism and transcriptome in NSCLC cells (121). Moreover, melatonin combined with USP7 inhibitor P5091 may enhance anti-cancer activity in p53 deficient NSCLC (122).

## Conclusion

This review has systematically summarized that various stress factors *in vivo* and *in vitro*, including hypoxia, oxidative stress, acidosis, glutamine and glucose deficiency, and other adverse factors, may activate the ER stress response to varying degrees during the progression of lung cancer. ER stress is extensively involved in the epithelial-mesenchymal transition, drug resistance, apoptosis, as well as immune regulation of lung cancer cells, and modulates multiple signaling pathways of lung cancer. Inhibitors targeting the UPR signaling pathway also participate in the bioactivities of tumors.

Our study reveals a crucial mechanistic foundation for enhancing immunotherapy, revealing how ER stress drives immunosuppression and identifying strategies to exploit ER stress-induced ICD and reverse immune evasion for combination therapies with checkpoint inhibitors. In addition, in-depth exploration highlights the potential for molecularly guided personalized therapy, emphasizing how tumor heterogeneity influences ER stress responses, paving the way for biomarker-stratified treatment using UPR activity markers.

Despite significant advances in understanding ER stress in lung cancer, several limitations and challenges remain. First, current knowledge relies heavily on preclinical models. Translating mechanistic insights on ER stress-induced EMT, apoptosis, autophagy, drug resistance, and immune modulation into effective clinical strategies requires robust validation in human studies. Second, the UPR is a dynamic network with significant crosstalk. The net outcome is highly context-dependent, influenced by stress intensity, duration, cell type, genetic background, and the tumor microenvironment. This complexity makes therapeutic targeting inherently challenging and prone to unintended consequences. Finally, the influence of specific lung cancer driver mutations and histological subtypes on ER stress responses and dependencies is underexplored. Future research needs to define context-specific UPR roles to enable biomarker-driven therapies.

In summary, targeting the regulatory molecules of ER stress and UPR in lung cancer may provide a new direction for tumor therapy. While due to the complex interactions between the structure of proteins involved in ER stress and signaling pathways, the off-target phenomenon of targeted agents is also a new challenge. With the accumulation and integration of multi-omics data, the treatment of lung cancer will be gradually addressed.
